# NtrC-dependent control of exopolysaccharide synthesis and motility in *Burkholderia cenocepacia* H111

**DOI:** 10.1371/journal.pone.0180362

**Published:** 2017-06-29

**Authors:** Yilei Liu, Martina Lardi, Alessandro Pedrioli, Leo Eberl, Gabriella Pessi

**Affiliations:** Department of Plant and Microbial Biology, University of Zurich, Zurich, Switzerland; Centre National de la Recherche Scientifique, Aix-Marseille Université, FRANCE

## Abstract

*Burkholderia cenocepacia* is a versatile opportunistic pathogen that survives in a wide variety of environments, which can be limited in nutrients such as nitrogen. We have previously shown that the sigma factor σ^54^ is involved in the control of nitrogen assimilation and virulence in *B*. *cenocepacia* H111. In this work, we investigated the role of the σ^54^ enhancer binding protein NtrC in response to nitrogen limitation and in the pathogenicity of H111. Of 95 alternative nitrogen sources tested the *ntrC* showed defects in the utilisation of nitrate, urea, L-citrulline, acetamide, DL-lactamide, allantoin and parabanic acid. RNA-Seq and phenotypic analyses of an *ntrC* mutant strain showed that NtrC positively regulates two important phenotypic traits: exopolysaccharide (EPS) production and motility. However, the *ntrC* mutant was not attenuated in *C*. *elegans* virulence.

## Introduction

The betaproteobacterium *Burkholderia cenocepacia* is an opportunistic pathogen that thrives in different environments, which can be limited in essential elements such as nitrogen [1, 2]. Bacterial adaptations to changes in nitrogen availability have been shown to be stringently regulated [3–7]. Enterobacteria respond to nitrogen starvation by activating the nitrogen regulatory response (Ntr) to facilitate N scavenging from alternative nitrogen sources. The Ntr system monitors the intracellular ratio of glutamine to α-ketoglutarate. Under nitrogen limiting conditions, the PII signal transduction proteins encoded by *glnB* and *glnK* are uridylylated and, by controlling the kinase and phosphatase activities of the regulator NtrB, regulate the transcription of nitrogen-regulated target genes [3, 6, 8, 9]. NtrB is a sensor kinase, and is part of the NtrBC two-component regulatory system. Under nitrogen limiting conditions, NtrB phosphorylates the response regulator NtrC, which then binds to DNA sequences in the promoter and together with the alternative sigma factor σ^54^ (or RpoN) activates transcription [10–12]. The sigma factor σ^54^ reversibly associates with the core RNA polymerase and recognizes its cognate promoter sequences via defined consensus sequences at positions –12 and –24 bp (relative to the transcription start site) [13]. The initiation of σ^54^-dependent transcription usually requires such an interaction with an enhancer binding protein (EBP). The specific protein involved varies depending on the respective environmental signals. NtrC is the EBP in the case of nitrogen starvation conditions [14, 15]. EBPs share a conserved modular structure, which consists of three domains: i) an amino terminal regulatory domain, ii) a central catalytic domain that belongs to the AAA+ superfamily of ATPases and is required for interaction with σ^54^, iii) a carboxy-terminal DNA-binding domain with a helix–turn–helix motif that is required for recognition of upstream activator sequences [16]. The EBP catalyzes ATP hydrolysis and thereby promotes conversion of the closed promoter into an open promoter complex from which transcription can proceed [16–20]. In enterobacteria, hexamers of phosphorylated NtrC bind to an upstream activator sequence (UAS), which is usually located 100–150 nucleotides upstream of the transcriptional start site [21, 22].

We have previously characterized the response of *B*. *cenocepacia* H111 to nitrogen starvation, a condition that is relevant during chronic lung infection [2, 23] and showed that nitrogen assimilation is largely dependent on the sigma factor σ^54^ [24]. Further phenotypic analysis of a σ^54^ mutant showed that this sigma factor is also important for other traits, which have been previously shown to play important roles during infection with *B*. *cenocepacia* [18, 25–30]: exopolysaccharide (EPS) production, biofilm formation and motility. The sigma factor σ^54^ is also required for H111 virulence in the *Caenorhabditis elegans* infection model [24].

Among the regulatory genes highly up-regulated under nitrogen starvation conditions was the two-component regulatory system NtrBC, suggesting that NtrC is the EBP of σ^54^ during nitrogen starvation [24]. In this study, the *ntrC* gene (I35_2149) was mutated in *B*. *cenocepacia* H111 and the derived NtrC regulon during nitrogen starvation was compared with the previously identified σ^54^ regulon. Phenotypic analysis of the *ntrC* mutant strain confirmed the transcriptomics data and showed that NtrC controls utilization of several nitrogen sources, EPS production and motility. While biofilm formation was partially compromised in the *ntrC* mutant, NtrC was not important for *C*. *elegans* virulence, suggesting that for this phenotype σ^54^ interacts with other EBPs or σ^54^ functions independently of EBPs.

## Material and methods

### Bacterial strains, media and growth conditions

The bacterial strains and plasmids used in this study are listed in S1 Table. *E*. *coli* and *B*. *cenocepacia* H111 cells were routinely grown in Luria broth (LB) [31] at 37°C using the following concentrations of antibiotics (in μg/ml): ampicillin (100 for *E*. *coli*), chloramphenicol (20 for *E*. *coli* and 80 for *B*. *cenocepacia*) and gentamycin (10 for both *E*. *coli* and *B*. *cenocepacia*). Cultures for RNA-Seq were grown first in AB minimal medium [32] using 10 mM sodium citrate as carbon source and 15 mM ammonium chloride (NH_4_Cl) as nitrogen source. Nitrogen starved conditions were created using AB minimal medium containing 0.3 mM NH_4_Cl. Cultures were grown in 500 ml Erlenmeyer flasks containing 100 ml medium on a shaker (200 rpm) at 37°C. For each strain or condition, the growth of three independent cultures was analysed.

### Construction of *B*. *cenocepacia* H111 mutant strains

Plasmid DNA from *E*. *coli* strains was obtained by using the QIAprep Spin Miniprep Kit (QIAGEN). To generate H111-*ntrC* (an insertional mutant in I35_2149, the ortholog of *B*. *cenocepacia* J2315 BCAL2222), a 508 bp internal fragment of I35_2149 was amplified with Ex Taq polymerase (TaKaRa) using primers ntrC_mut_new_F and ntrC_mut_new_R (see S1 Table). The PCR product was cloned into pGEM^®^-T Easy Vector (Promega) and then sub-cloned into pSHAFT2 [33] as a *Not*I fragment to generate pSHAFT2-*ntrC*. The resulting plasmid was mobilized into *B*. *cenocepacia* H111 wild type by triparental mating. Correct genomic integration was verified by PCR using oligos ntrC_Comp_F and pSHAFT_R. To complement H111-ntrC, the complete I35_2149 ORF was amplified with Phusion High-Fidelity DNA polymerase (Thermo Fisher Scientific) using oligos ntrC_Comp_F and ntrC_Comp_R (S1 Table). The PCR product was first cloned into pGEM^®^-T Easy Vector, digested with *Eco*RI, and then cloned into *Eco*RI-digested pBBR1MSC-5 (Gm^R^) to create the complementing plasmid pBBR1-*ntrC*. The sequence of the I35_2149 ORF in the complementation vector was verified by DNA sequencing. The complementing plasmid was mobilized into H111-*ntrC* by triparental mating to generate the complemented strain.

### RNA-Seq and data analysis

H111 wild-type and mutant strains were grown to exponential phase in AB minimal medium containing 15 mM NH_4_Cl, and washed twice in AB minimal medium without NH_4_Cl. The cells were further incubated for 1 hour under nitrogen starvation conditions. Three independent RNA-Seq experiments were performed from three independent biological replicates. Total RNA was extracted using a modified hot acid phenol protocol [34]. The complete removal of genomic DNA using RQ1 RNase-Free DNase (Promega) was verified by a PCR reaction with 40 cycles. Samples were further purified using the RNeasy Mini Kit (QIAGEN) and RNA quality was checked using RNA Nano Chips (Agilent 2100 Bioanalyzer). A total of 150 ng RNA was used for cDNA synthesis and library preparation using the Ovation® Complete Prokaryotic RNA-Seq Library System from NuGEN. The cDNA libraries were analyzed and quantified by capillary electrophoresis using D1000 ScreenTape from Agilent (size range 100–800 bp). Illumina single-end sequencing was performed on a HiSeq2500 instrument. The sequence reads were processed and then mapped to the recently finished H111 genome sequence (accession no. HG938370, HG938371, and HG938372) [35] using CLC Genomics Workbench v7.0 (CLC bio) allowing up to 2 mismatches per read. The mapped reads were analysed using the DESeq software [36]. The RNA-Seq raw data files are accessible through the GEO Series accession number GSE95607.

Functional analysis was based on the EggNOG annotation that is available for *B*. *cenocepacia* J2315 [37] and was performed as previously described [24]. Distribution of categories was determined using a Fischer test with the online quick calculator of GraphPad (p-value < 0.01).

### Phenotypic analysis of mutant strains

The utilization of nitrogen sources by H111 wild-type and *ntrC* mutant strains was assessed using Biolog PM3B plates according to the manufacturer’s instructions as described previously [38]. After inoculation, the plates were incubated at 37°C for 24 hours before the OD_590_ of each well was measured by a plate-reader (TECAN). The assay was performed on biological duplicate cultures for each strain. The OD_590_ of these was taken to calculate the average values and standard deviation. The 18 differentially utilized N sources were determined using the following criteria: i) ratio of average final OD_590_ (wild-type vs. mutant or vice-versa) > 2; ii) the higher OD_590_ average value > 0.3; iii) both standard deviations (of wild-type and mutant) < 0.1.

The assimilation of nitrogen sources was also assayed by observing growth in modified AB minimal medium, where 15 mM NH_4_Cl was replaced by other nitrogen sources at concentrations according to the number of N atoms in the molecule. The cells were incubated in 16 ml medium with a starting OD_600_ of 0.05 for 4 days at 37°C with shaking at 40 rpm. The final OD_600_ of the independent biological triplicate cultures for each strain was used to calculate the mean and the standard deviation. For each nitrogen source, all 3 strains (wild-type, *ntrC* mutant and the complemented mutant) were tested in parallel.

EPS production was assessed on modified YEM medium plates (0.4% mannitol, 0.05% yeast extract, 1.5% agar [39]). All 3 strains to be tested were streaked in parallel on the YEM plate to eliminate variations. Independent biological triplicate cultures for each strain were tested. Images were taken for evaluation after 24 hours incubation at 37°C.

Biofilm formation was quantified in a microtiter-plate assay as described by Huber *et al*. [40]. For each condition, all 3 strains were tested in parallel in one plate and independent biological triplicate cultures for each strain were tested. Each culture was tested in at least 4 wells in one plate. The biofilm index was calculated for each well. The mean and the standard deviation of all the biofilm index data for each strain were used to make the histogram. A two-tailed unpaired Student’s t test was used to determine the significance between either mutant or complement and wild type.

Swarming and swimming activity were tested in the same way as previously described [41] with the following modifications: swarming and swimming assays were carried out on AB minimal medium plates containing 10 mM glucose as carbon source and 0.1% casamino acids, solidified with 0.4% and 0.2% agar, respectively. Independent biological triplicate cultures of each strain were tested. Swarming assays were carried out with minor modifications. In detail, swarming plates were freshly prepared and air-dried for half an hour under the laminar flow after solidification. Pre-culture cells were washed twice with AB minimal medium and adjusted to OD_600_ 0.5. Then 3 μl of each culture was inoculated at the center of a plate. The plates were incubated in humid conditions at 37°C for 5 days. The colony swarming diameter for each plate was measured. For both swarming and swimming assays, at least independent biological triplicate cultures of each strain were tested and the 3 strains were always tested in parallel for each assay. To normalize the data, for each assay the average diameter of the wild type was set to 100% and used as a reference. The *ntrC* mutant and complement strain values were shown as a percentage of this reference value. Swimming and swarming motility were statistically analysed using a two-tailed unpaired Student’s t test, with either the mutant or complemented mutant compared to the wild-type strain.

Pathogenicity tests using *Caenorhabditis elegans* Bristol N2 strain and *Galleria mellonella* were performed as previously described [24] and [38], respectively. For *C*. *elegans*, the number of synchronized L1 larvae was recorded after seeding with the bacteria into each well of a 96-well plate to test. After 48 hours co-incubation at 20°C, the worms were scored according to their developmental stages and the reduction in the total number of surviving larvae was regarded as “dead”. The assays using both infection models were carried out in triplicate with all 3 strains tested in parallel.

### qPCR analysis

The qPCR analysis was carried out using Brilliant III Ultra-Fast SYBR® Green QPCR Master Mix (Agilent, Switzerland) and an Mx3000P instrument (Agilent, Switzerland). The cDNA was prepared from an independent biological replicate (of both wild-type and *ntrC* mutant strains) as previously described [42]. Each PCR reaction was run in triplicate with 3 dilutions of cDNA (15, 7.5 and 3.75 ng) using 15 μl 2x Brilliant III Ultra-Fast SYBR® Green QPCR Master Mix, and 5 μM of individual primers in a total volume of 30 μl. Fold-changes in transcription and the standard deviation of 9 sample dilutions were calculated using the ΔΔ CT method [43]. The primary σ factor gene *rpoD* was used as a reference for normalization. All the primers used are listed in S1 Table.

## Results

### Construction and growth analysis of a *B*. *cenocepacia* H111 *ntrC* mutant and a complemented derivative

To study the role of the σ^54^ activator protein NtrC in response to nitrogen limitation, an H111 *ntrC* mutant and a complemented mutant were constructed (S1 Table). The gene I35_2149, an ortholog of *B*. *cenocepacia* strain J2315 gene BCAL2222, was chosen since it showed 55% amino acid identity and 67% amino acid similarity to NtrC of *Escherichia coli*. Furthermore, its transcription was found to be significantly up-regulated by nitrogen starvation and significantly down-regulated in the absence of the alternative sigma factor σ^54^, strongly suggesting that NtrC is the EBP of σ^54^ in nitrogen limited environments [44]. Similar to other bacteria, the gene is located in an operon downstream of the gene coding for its potential sensor kinase NtrB and the gene *glnA* which encodes a glutamine synthetase. While the *ntrC* mutant grew at a similar rate and to the same final optical density (i. e. OD_600_ of 4) as the wild type in LB, the mutant had a moderate growth delay in AB minimal medium containing citrate as carbon source (S1 Fig). The complemented *ntrC* mutant displayed a growth defect in both media, but was able to eventually reach the same final optical density (OD_600_) as the wild type (data not shown). In contrast, on LB or AB minimal medium agar plates, the growth of the wild type and the complemented *ntrC* mutant was indistinguishable after 24 and 48 hours of incubation, respectively. The growth defect of the complemented mutant may have been due to the over-expression of *ntrC* from the pBBR1MCS-5 plasmid. Our qPCR result showed that when the cells were grown in LB until stationary phase, *ntrC* transcription in the complemented strain was 162 fold higher than the wild-type strain (as determined by qPCR).

### NtrC affects the utilization of alternative nitrogen sources

**Fig 1:**
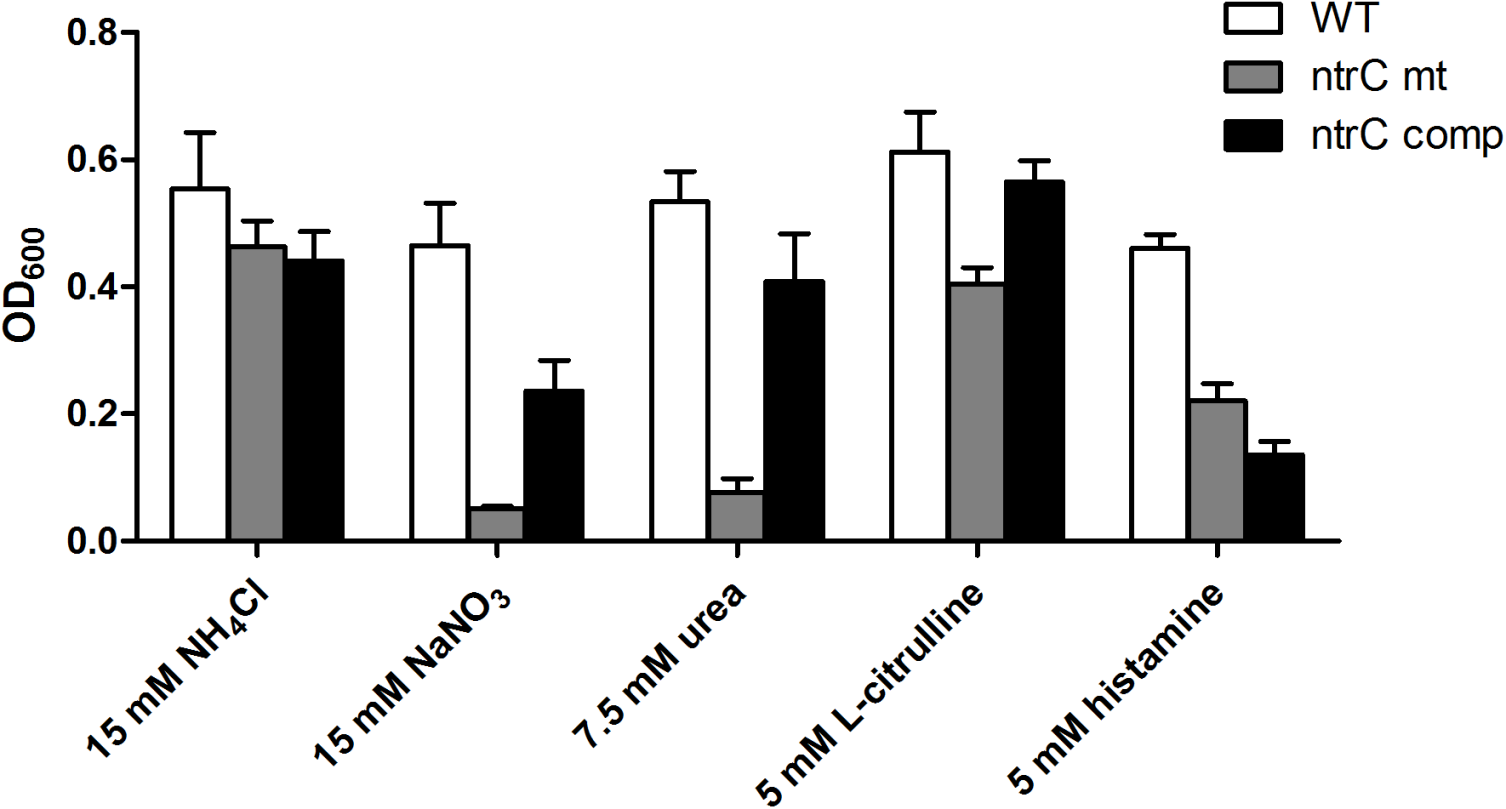
Nitrogen sources differentially utilised by the wild-type, *ntrC* mutant and the complemented strains. Cells were grown at 37°C for 4 days with slow agitation in order to synchronize the growth. 3 independent cultures of each strain were tested with each N source. Columns and error bars indicate the average and the standard deviation of final OD_600_, respectively.

The ability of the wild-type strain and the *ntrC* mutant to utilize 95 different nitrogen sources was examined using Biolog plate assays (S2 Table). The utilization of 18 N sources was found to be affected by NtrC. In 7 cases (nitrate, urea, L-citrulline, acetamide, DL-lactamide, allantoin and parabanic acid), the *ntrC* mutant was compromised in substrate utilization. In contrast, with 11 N sources (biuret, L-cysteine, L-isoleucine, L-methionine, hydroxylamine, methylamine, ethylamine, ethylenediamine, N-acetyl-D-galactosamine, uric acid, ε-amino-N-caproic acid) the *ntrC* mutant showed enhanced growth compared to the wild-type. The utilization of selected N sources was also tested using a different experimental setup (Fig 1). When incubated at 37°C in AB minimal medium containing 15 mM ammonium as N source, the H111 wild-type, *ntrC* mutant and *ntrC* complemented strains grew at similar rates. Consistent with the Biolog results, the *ntrC* mutant was unable to use nitrate or urea as sole N source. However, in the case of L-citrulline and histamine, growth was only mildly affected in the mutant compared with the wild-type. Heterologous complementation with *ntrC* partially rescued growth of mutant with most of the N sources tested, except for histamine.

### Mapping the NtrC regulon in *B*. *cenocepacia* H111

**Fig 2:**
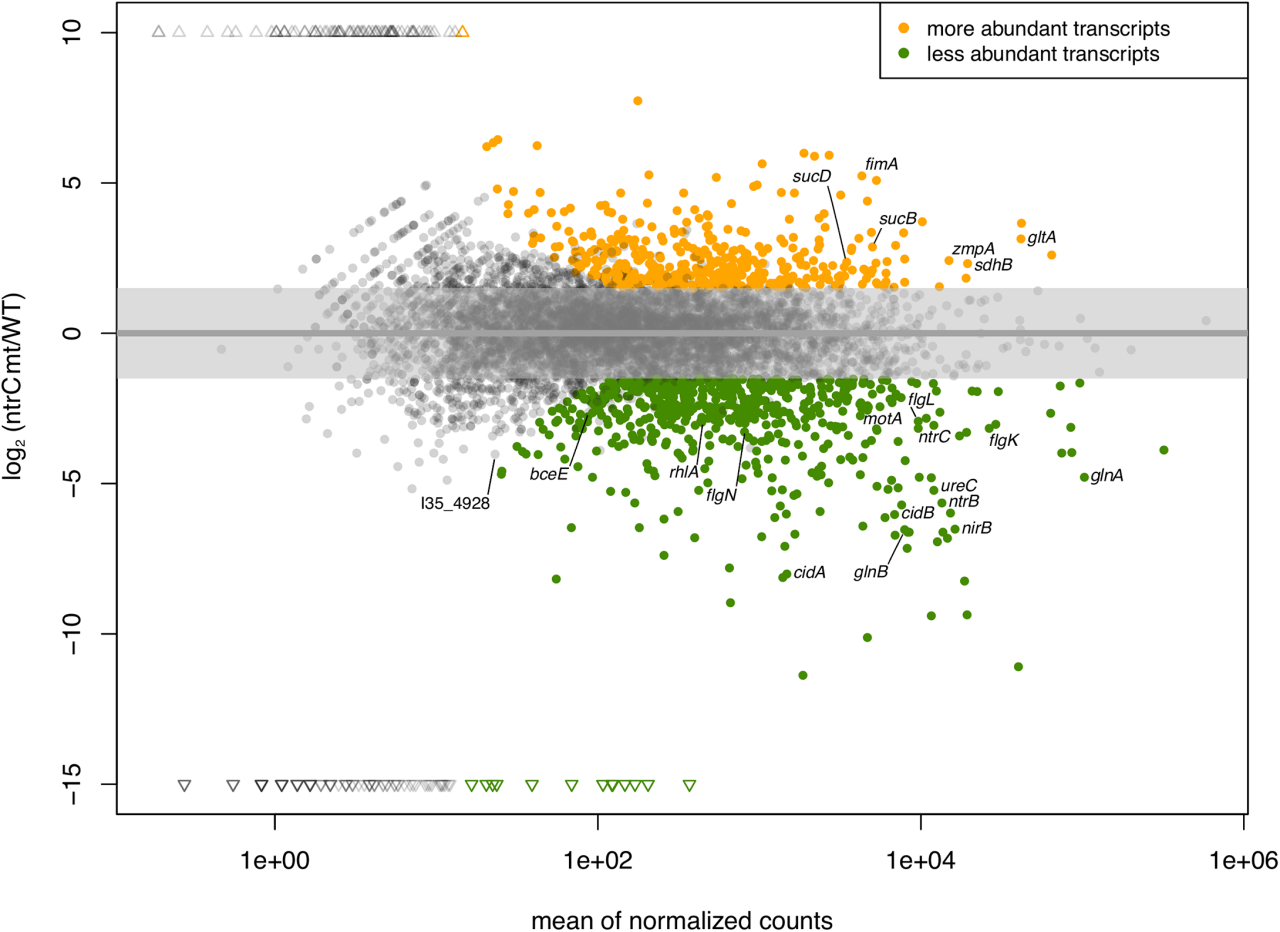
Differential transcript expression in the *ntrC* mutant compared to the wild type. MA plot showing the log_2_ fold change in transcript expression of *B*. *cenocepacia* H111 wild type and *ntrC* mutant strains grown under nitrogen limited conditions. The top regulated genes (p-value < 0.001, absolute log_2_ (Fold Change) > 1.5) are shown in color: genes with increased transcription in the *ntrC* mutant compared to the wild type are indicated in orange, down-regulated genes in green. The names of the genes of particular interest are labelled.

To identify genes controlled by NtrC, RNA-Seq was performed on the *ntrC* mutant and the wild-type strain grown under nitrogen limiting conditions. For this, RNA was extracted from independent biological triplicate cultures of wild-type and *ntrC* mutant cells grown first in AB minimal medium containing 15 mM NH_4_Cl until exponential phase (OD_600_ = 0.5, S1 Fig) and then shifted to nitrogen limiting conditions (0.3 mM NH_4_Cl) for one hour (shift experiment). Among the 150 top ranked differentially expressed genes (DESeq analysis p-value < 10^−20^, absolute log_2_(Fold Change) > 2.3) (Fig 2 and Table 1), 123 genes (82%) showed decreased transcription in the *ntrC* mutant. Genes involved in nitrogen assimilation such as the glutamine synthetase (*glnA*, I35_2151) and one of the two PII sensor proteins (*glnB*, I35_2936) showed 4.8- and 6.5- fold (log_2_ fold change) reduced transcription, respectively, in the *ntrC* mutant. In line with the inability of the *ntrC* mutant to grow with urea or nitrate as the sole nitrogen source, the genes required for urea (I35_0765–0775 and I35_7283–7286) and nitrate (I35_5545–5548) transport and utilization displayed down-regulated transcription in the *ntrC* mutant. Sixty-five percent of the genes found to be activated by NtrC have previously been shown to have increased transcription under nitrogen limited conditions [24], suggesting that *B*. *cenocepacia* NtrC plays a major role in the control of gene transcription during nitrogen deprivation. Among the top 150 genes significantly differentially transcribed in the *ntrC* mutant (Table 1), 95% were also regulated by σ^54^, suggesting that, as is the case in other bacteria, NtrC is the EBP of σ^54^ required for activating transcription of genes under nitrogen limiting conditions (see below). Among the common target genes of σ^54^ and NtrC, many were potentially involved in nitrogen metabolism: the urease and nitrate reductase gene clusters described above, a cluster coding for proteins containing a transglutaminase-like domain (I35_5080–83), a xanthine dehydrogenase (I35_0688–0689), a gene cluster containing a urate oxidase (I35_1963–64), several genes coding for components of ABC transporters (I35_0158, I35_4451, I35_4872–73, I35_5228–29, I35_5552, I35_5645, I35_6515, I35_7109–10, I35_7737–40), and an alanyl-alanine dipeptidase and its associated transporter (I35_5644–46). Moreover, other genes showed σ^54^ –NtrC–dependent transcription, including *cidAB* (I35_3288–89) coding for a putative holin and anti-holin system, I35_4672–73 encoding a poly-beta-hydroxyalkanoate (PHB) depolymerase and a glutathione-S-transferase, and I35_4766 (an ortholog of BCAM0853), which is part of the *bce*-I cluster that encodes the main *B*. *cenocepacia* EPS cepacian. We found several transcriptional regulators with decreased transcription in the *ntrC* mutant, including two genes coding for an ethanolamine operon regulatory protein (I35_0068 and I35_7815), I35_1967, *ntrB*, I35_4176, I35_4535, I35_4653, *nasT* (I35_5551), I35_5643, I35_5874 and I35_6218. An analysis of the categories associated with the top 150 NtrC-regulated genes, revealed that, beside category E (amino acid metabolism and transport), category N (motility) is over-represented among the genes positively regulated by NtrC (S2 Fig). In fact, Table 1 contains several genes involved in flagellar biosynthesis (I35_3089–90 and I35_3103) and rotation (*motAB*, I35_0133–34), and a gene (I35_0139) involved in chemotaxis.

**Table 1:** List of the 150 genes with statistically significant differential expression, comparing the expression profile of the *ntrC* mutant with the profile of the wild type (DE-Seq analysis, p-value < 10^−20^, absolute log_2_(Fold Change) > 2.3).

Class	Locus ID^a^	Orthologs J2315^b^	Description^a^	Gene name	Log_2_ ntrC vs wt^c^
Amino acid metabolism and transport	I35_0158	BCAL0151	Leucine-, isoleucine-, valine-, threonine-, and alanine-binding protein		-3.6
	**I35_0767**	**BCAL3106**	**Urease alpha subunit**	***ureC***	**-5.2**
	**I35_0769**	**BCAL3104**	**Urease gamma subunit**	***ureA***	**-4.4**
	**I35_0772**	**BCAL3102**	**Urea ABC transporter, ATPase protein**	***urtD***	**-4.2**
	**I35_0774**	**BCAL3099**	**Urea ABC transporter, permease protein**	***urtB***	**-5.9**
	**I35_0775**	**BCAL3098**	**Urea ABC transporter, urea binding protein**		**-4.8**
	I35_1846	BCAL1926	Homoserine dehydrogenase	*hom*	2.8
	**I35_2151**	**BCAL2224**	**Glutamine synthetase type I**	***glnA***	**-4.8**
	**I35_2936**	**BCAL0729**	**Nitrogen regulatory protein P-II**	***glnB***	**-6.5**
	I35_4872	BCAM0952	Putrescine transport ATP-binding protein	*potA*	-3.1
	I35_4873	BCAM0953	ABC transporter, periplasmic spermidine putrescine-binding protein	*potD*	-3.1
	**I35_5082**	**BCAM1235**	**Protein containing transglutaminase-like domain**		**-9.4**
	**I35_5083**	**BCAM1236**	**Large protein containing transglutaminase-like domain**		**-6.0**
	I35_5229	BCAM1377	ABC-type spermidine/putrescine transport systems,ATPase components		4.0
	I35_7109	BCAS0112	ABC-type arginine/histidine transport system,permease component		-5.2
	I35_7110	BCAS0113	Histidine ABC transporter, ATP-binding protein	*hisP*	-3.8
	**I35_7286**	**BCAS0272**	**Urea carboxylase**		**-6.0**
	I35_7447	BCAS0409	Zinc metalloprotease	*zmpA*	2.4
	I35_7737	BCAS0574	Glutamate transport ATP-binding protein		-4.3
	I35_7739	BCAS0576	amino acid ABC transporter, permease protein		na(m)
	I35_7740	BCAS0577	ABC-type amino acid transport, periplasmic component		-10.1
	**I35_7857**	**BCAS0734**	**Pyridine nucleotide-disulphide oxidoreductase**		**-6.8**
	**I35_7858**	**BCAS0735**	**Beta-ureidopropionase**		**-6.9**
	I35_7874	BCAS0751	Gamma-glutamyltranspeptidase		-4.8
Carbohydrate metabolism and transport	**I35_1964**	**BCAL2040**	**Chitooligosaccharide deacetylase;** **putative uricase**		**-3.2**
	**I35_2891**	**BCAL0782**	**Beta-hexosaminidase**		**4.7**
Cell wall, membrane, envelope biogenesis	I35_0402	BCAL3473	Outer membrane protein (porin)		-4.2
	I35_0812	BCAL3057	Soluble lytic murein transglycosylase		3.2
	I35_3042	BCAL0624	Outer membrane protein (porin)		-6.4
	**I35_3288**	**BCAL3508**	**CidA-associated membrane protein**	***cidB***	**-6.0**
	I35_4371	BCAM0478	Glucosamine—fructose-6-phosphate aminotransferase [isomerizing]	*glmS2*	-5.0
	**I35_4934**	**BCAM1015**	**Outer membrane protein (porin)**		**-4.0**
	**I35_5228**	**BCAM1376**	**Porin, Gram-negative type**		**5.6**
	I35_5644	BCAM1769	D-alanyl-D-alanine dipeptidase	*ddpX*	-4.5
	**I35_5658**	**BCAM1780**	**Lipoprotein**	***nlpD***	**-3.5**
	I35_7271	BCAS0256	Outer membrane protein (porin)		-3.2
Cell motility	**I35_0133**	**BCAL0126**	**Flagellar motor rotation protein**	***motA***	**-2.7**
	**I35_0134**	**BCAL0127**	**Flagellar motor rotation protein**	***motB***	**-3.1**
	I35_0139	BCAL0132	Chemotaxis protein methyltransferase	*cheR*	-2.5
	**I35_3089**	**BCAL0577**	**Flagellar hook-associated protein**	***flgL***	**-2.9**
	**I35_3090**	**BCAL0576**	**Flagellar hook-associated protein**	***flgK***	**-3.2**
	**I35_3103**	**BCAL0561**	**Flagellar biosynthesis protein**	***flgN***	**-3.3**
Coenzyme metabolism	I35_1039	BCAL1047	Pyridoxal kinase	*pdxY*	-2.4
	**I35_1623**	**BCAL1711**	**Component of cobalt chelatase involved in B12 biosynthesis**	***cobN***	**3.5**
	**I35_5550**	**BCAM1687**	**Uroporphyrinogen-III methyltransferase**		**-11.4**
Defense mechanism	I35_3251	BCAL0420	Type I restriction-modification system, restriction subunit R		2.9
Energy production and conversion	I35_1414	BCAL1516	Dihydrolipoamide succinyltransferase component	*sucB*	2.9
	**I35_1735**	**BCAL1819**	**Oxidoreductase (flavoprotein)**		**-5.4**
	**I35_2213**	**BCAL2287**	**Fumarate hydratase class I**		**3.2**
	I35_2268	BCAL2344	NADH ubiquinone oxidoreductase chain A	*nuoA*	2.4
	**I35_2713**	**BCAL0957**	**Succinyl-CoA ligase [ADP-forming] alpha chain**	***sucD***	**2.4**
	I35_3124	BCAL0541	Aminobutyraldehyde dehydrogenase		-2.7
	**I35_4890**	**BCAM0970**	**Succinate dehydrogenase iron-sulfur protein**	***sdhB***	**2.3**
	**I35_4892**	**BCAM0972**	**Citrate synthase**	***gltA***	**3.1**
	**I35_5545**	**BCAM1683**	**Assimilatory nitrate reductase large subunit**		**-6.7**
	**I35_5546**	**BCAM1684**	**Nitrite reductase [NAD(P)H] small subunit**		**-6.8**
	**I35_5547**	**BCAM1685**	**Nitrite reductase [NAD(P)H] large subunit**	***nirB***	**-6.5**
	**I35_7856**	**BCAS0733**	**Dihydropyrimidine dehydrogenase [NADP+]**		**-5.1**
Inorganic ion transport and metabolism	**I35_2600**	**BCAL2740**	**HoxN/HupN/NixA family nickel/cobalt transporter**	***hoxN***	**-4.7**
	**I35_4451**	**BCAM0556**	**Dibenzothiophene desulfurization enzyme B**		**na(m)**
	**I35_5230**	**BCAM1378**	**ABC Fe3+ siderophore transporter** **inner membrane subunit**		**3.8**
	**I35_5548**	**BCAM1686**	**Nitrate/nitrite transporter**		**-6.6**
	**I35_5552**	**BCAM1689**	**Nitrate ABC transporter, nitrate-binding protein**		**-4.2**
	I35_5646	BCAM1771	Dipeptide transport system permease protein	*dppB*	-3.2
	I35_5693	BCAM1814	Cyclohexanone monooxygenase		-2.8
	**I35_7283**	**BCAS0269**	**Urea carboxylase-related ABC** **transporter, periplasmic protein**		**na(m)**
Lipid metabolism	**I35_4672**	**BCAM0774**	**Poly-beta-hydroxyalkanoate depolymerase**		**-6.6**
Nucleotide metabolism and transport	I35_0495	BCAL3380	Allantoicase		-3.5
	I35_0689	BCAL3172	Xanthine dehydrogenase, molybdenum binding subunit	*xdhB*	-3.1
	**I35_7854**	**BCAS0731**	**Dihydropyrimidinase**	***dhT***	**-3.7**
	**I35_7855**	**BCAS0732**	**Putative pyrimidine permease in reductive pathway**		**-3.8**
Post translational modifications	**I35_0765**	**BCAL3108**	**Urease accessory protein**	***ureF***	**-7.1**
	**I35_0766**	**BCAL3107**	**Urease accessory protein**	***ureE***	**-7.8**
	**I35_2591**	**BCAL2731**	**ATP-dependent Clp protease adaptor protein**	***clpS***	**-2.6**
	**I35_2821**	**BCAL0849**	**Putative lipoprotein**		**3.1**
	I35_3125	BCAL0540	ATP-dependent protease domain protein		-4.1
	**I35_4673**	**BCAM0775**	**Glutathione S-transferase**		**-5.3**
	I35_5615	BCAM1744	Extracellular protease precursor		-2.3
Secondary structures	I35_2997	BCAL0668	Dienelactone hydrolase and related enzymes		-2.6
Transcription and signal transduction	**I35_0068**	**BCAL0066**	**Ethanolamine operon regulatory protein**		**-5.7**
	**I35_0200**	**BCAL0209**	**Histone acetyltransferase HPA2**		**-5.1**
	I35_1574	BCAL1663	Serine protein kinase	*prkA*	-4.0
	**I35_1967**	**BCAL2043**	**Transcriptional regulator, GntR family**		**-5.9**
	**I35_2150**	**BCAL2223**	**Nitrogen regulation protein NR(II)**	***ntrB***	**-5.6**
	I35_3013	BCAL0652	EAL domain protein		-7.2
	I35_4176	BCAM0176	Transcriptional regulator, AsnC family		-3.6
	I35_4382	BCAM0489	Mercuric resistance operon regulatory protein		-3.6
	I35_4535	BCAM0639	Two-component response regulator		-6.5
	I35_4613	BCAM0715	Signal transduction histidine kinase		3.5
	I35_4653	BCAM0754	Transcriptional regulator, TetR family		-3.6
	**I35_5551**	**BCAM1688**	**Response regulator**	***nasT***	**-8.1**
	I35_5643	BCAM1768	Transcriptional regulators		-6.1
	I35_5806	BCAM1975	Ethanolamine operon regulatory protein		-4.8
	**I35_5874**	**BCAM2039**	**Two-component response regulator**		**-5.0**
	**I35_6218**	**BCAM2327**	**Transcriptional regulator**		**-6.7**
Translation	I35_2322	BCAL2395	Cytoplasmic axial filament protein CafA and Ribonuclease G	*cafA*	-2.5
	I35_2859	BCAL0812	Ribosome hibernation protein	*yhbH*	-3.9
Others	I35_0339	BCAL0348	Uncharacterized protein	*impA*	3.4
	**I35_0341**	**BCAL0350**	**Hypothetical protein**		**2.6**
	I35_0688	BCAL3173	Xanthine dehydrogenase, iron-sulfur cluster and FAD-binding subunit A	*xdhA*	-3.0
	**I35_0743**		**Hypothetical protein**		**2.9**
	**I35_0963**	**BCAL2904**	**Hypothetical protein**		**-2.7**
	**I35_1398**	**BCAL1500**	**Transposase and inactivated derivatives**		**-5.2**
	I35_1575	BCAL1664	Hypothetical protein		-3.4
	I35_1576	BCAL1665	SpoVR-like protein		-3.0
	I35_1588	BCAL1677	Type 1 fimbriae major subunit	*fimA*	5.2
	**I35_1734**	**BCAL1818**	**Zn-dependent hydrolases, including glyoxylases**		**-6.6**
	**I35_1736**		**Oxidoreductase (flavoprotein)**		**-5.2**
	**I35_1739**	**BCAL1822**	**Putrescine transport system permease protein**	***potH***	**3.9**
	**I35_1872**	**BCAL1952**	**Hypothetical protein**		**4.4**
	I35_1881	BCAL1961	Ankyrin repeat protein		-2.5
	**I35_1963**	**BCAL2039**	**Urate oxidase**		**-3.4**
	I35_2376	BCAL2448	Phenazine biosynthesis protein PhzF like		3.1
	I35_2564	BCAL2703	Branched-chain amino acid transport ATP-binding protein		-2.4
	I35_3012	BCAL0653	Hypothetical protein		-4.3
	I35_3082	BCAL0584	Outer membrane porin		-3.3
	**I35_3289**	**BCAL3509**	**Holin-like protein**	***cidA***	**-8.0**
	I35_4192	BCAM0193	Hypothetical protein		6.0
	I35_4193	BCAM0194	Hypothetical protein		5.9
	I35_4195	BCAM0196	Hypothetical protein		4.7
	I35_4401	BCAM0507	Uncharacterized protein conserved in bacteria		-3.5
	I35_4471	BCAM0576	Hypothetical protein		-3.4
	I35_4651	BCAM0752	Hydrolase-related protein		-3.6
	I35_4652	BCAM0753	Hypothetical protein		-3.9
	I35_4669	BCAM0770	Hypothetical protein		-7.4
	**I35_4766**	**BCAM0853**	**Transposase and inactivated derivatives**		**-4.1**
	I35_4943	BCAM1098	MutT/nudix family protein		-2.7
	**I35_5080**	**BCAM1233**	**Protein containing domains DUF404, DUF407**		**-8.2**
	**I35_5081**	**BCAM1234**	**Protein containing domains DUF403**		**-9.4**
	I35_5153	BCAM1304	Phage-related protein		-2.4
	I35_5345	BCAM1491	Hypothetical protein		-4.8
	**I35_5549**		**Hypothetical protein**		**-11.1**
	I35_5645	BCAM1770	Dipeptide-binding ABC transporter, periplasmic component		-4.8
	I35_5655	BCAM1777A	Hypothetical protein		-3.6
	I35_5695	BCAM1816	Hypothetical protein		-2.7
	I35_5683	BCAM1804	Methyl-accepting chemotaxis protein		-2.9
	I35_5753	BCAM1927	Membrane-fusion protein		-3.1
	**I35_6093**	**BCAM2207**	**Hypothetical protein**		**-2.5**
	I35_6098	BCAM2209	Hypothetical protein		-3.0
	I35_6344	BCAM2444	Conserved domain protein		-4.1
	I35_6461	BCAM2564	Aerotaxis sensor receptor protein		-4.9
	I35_6515	BCAM2618	Histidine ABC transporter, histidine-binding periplasmic protein	*hisJ*	-4.4
	I35_6573	BCAM2679	Hypothetical protein		-9.0
	I35_6574	BCAM2680	Putative exported protein		-3.6
	I35_6579		Hypothetical protein		-4.6
	I35_7149		Hypothetical protein		-3.7
	I35_7282	BCAS0267a	3',5'-cyclic-nucleotide phosphodiesterase		-4.7
	I35_7285	BCAS0271	Urea carboxylase-related aminomethyltransferase		na(m)
	I35_7735	BCAS0571	Salicylate hydroxylase		-4.5
	I35_7815		Ethanolamine operon regulatory protein		-4.2

^a^Nomenclature and description according to GenBank file (accession no. HG938370, HG938371, and HG9383729).

^b^Orthologs were identified as described in the Material and Methods section.

^c^Fold change (FC) of transcription, comparing ntrC mutant with wild type grown under nitrogen limited conditions.

na, not applicable because the read number in the mutant is equal to 0.

all the genes with an rpoN box in the promoter region are indicated in bold.

Among the 27 genes with increased transcription in the *ntrC* mutant (Table 1) we found *fimA* (I35_1588 encoding a type I fimbria), *zmpA* (I35_7447 coding for a zinc metalloprotease), two genes in a type VI secretion system cluster (I35_0339 and I35_0341), I35_3251 coding for a subunit of a type I restriction-modification system and several genes involved in the Tricarboxylic Acid Cycle (TCA) cycle (*sucB*, *sucD*, *sdhB*, *gltA* and I35_2213 coding for a fumarase). Using a less stringent p-value threshold (DESeq analysis p-value < 10^−13^, absolute log_2_(Fold Change) > 1.5; S3 Table) additional genes involved in nitrogen metabolism and motility were identified as NtrC-regulated. Additionally, two genes in the cepacian clusters *bce*I (I35_4767 and I35_4772), the gene *rhlA* (I35_6233) coding for an enzyme catalysing the first step in biosynthesis of rhamnolipids, I35_1797 coding for the RNA binding protein Hfq and several protease-encoding genes (*clpS*, *clpX*, *lon*, *ybbK*) showed decreased transcription in the *ntrC* mutant. The NtrC-dependent transcription of genes involved in cepacian, rhamnolipid biosynthesis and cell motility was validated by an independent qPCR analysis (Table 2).

**Table 2:** Validation of selected RNA-Seq results by qPCR.

Locus ID^a^	J2315 orthologs^b^	Description^a^	Gene name	Log_2_ FC MT vs WT^c^	Log_2_ FC MT vs WT^d^
I35_0767	BCAL3106	Urease alpha subunit	*ureC*	-5.0	-5.2
I35_2151	BCAL2223	Glutamine synthetase	*glnA*	-3.6	-4.8
I35_4771	BCAM0858	Polysaccharide export lipoprotein	*bceE*	-1.2	-2.6
I35_4928	BCAM1009	O-antigen acetylase		-1.1	-4
I35_6233	BCAM2340	3-(3-hydroxyalkanoyloxy) alkanoic acids synthase	*rhlA*	-1.6	-2.9
I35_0133	BCAL0126	Flagellar motor rotation protein	*motA*	-0.9	-2.7
I35_3089	BCAL0577	Flagellar hook-associated protein	*flgL*	-2.4	-2.9
I35_3103	BCAL0561	Flagellar biosynthesis protein	*flgN*	-1.8	-3.3

^a^Nomenclature and description according to GenBank file (accession no. HG938370, HG938371, and HG938372).

^b^Orthologs were identified as described in the Material and Methods section.

^c^Fold change (FC) of transcription determined by qPCR, comparing the *ntrC* mutant (MT) with the wild type (WT) grown in AB minimal medium with a shift experiment. The standard deviation is less than 10% of the fold change.

^d^Fold change (FC) of transcription determined by RNA-Seq, comparing the *ntrC* mutant (MT) with the wild type (WT) grown in AB minimal medium (shift experiment).

### NtrC regulates EPS production but has only a slight effect on biofilm formation

**Fig 3:**
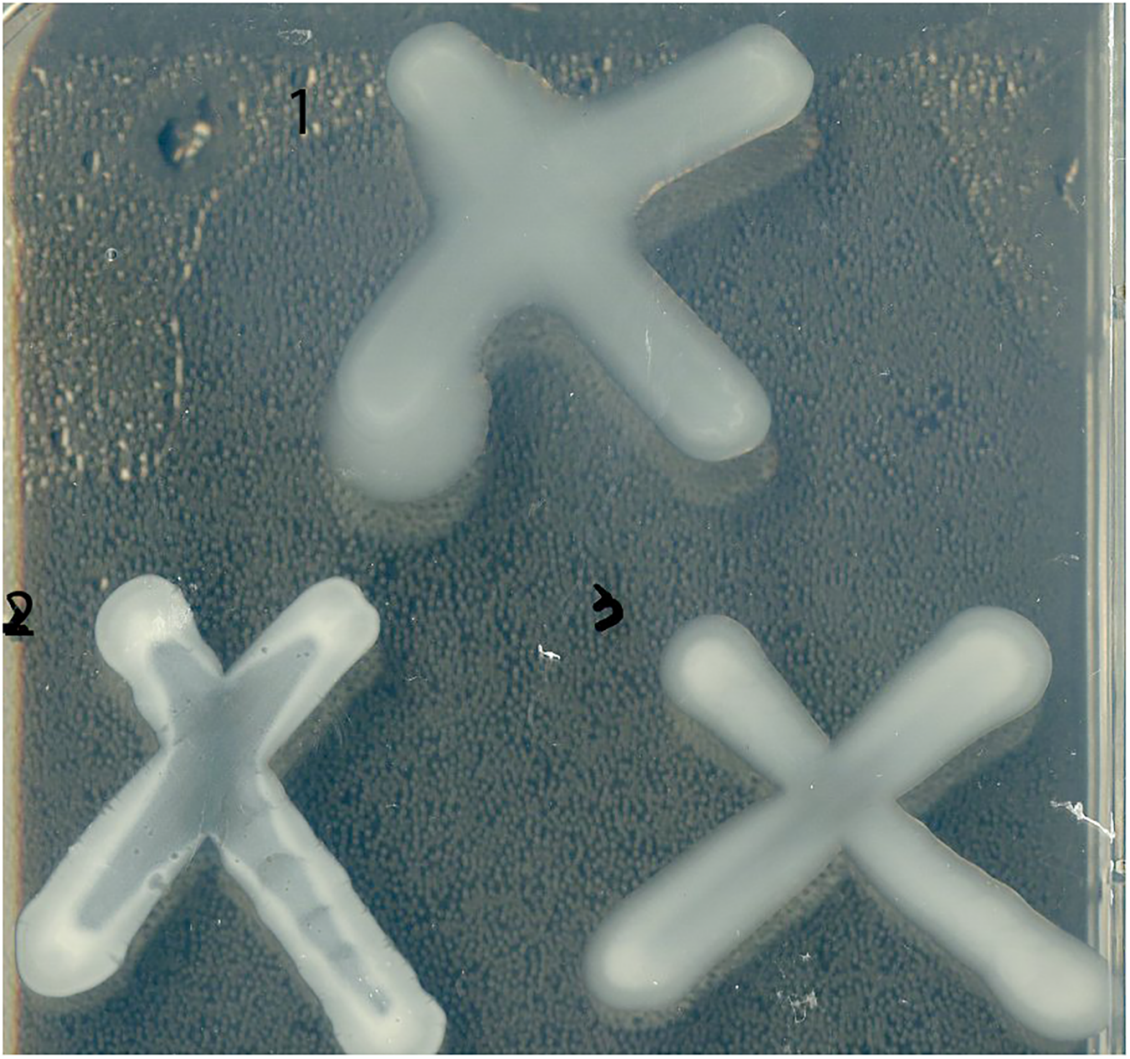
NtrC–dependent EPS production. EPS production in the wild type (1), the *ntrC* mutant (2) and the complemented strain (3) was tested on YEM plates. Three independent biological replicates were tested; the result of one is shown here.

**Fig 4:**
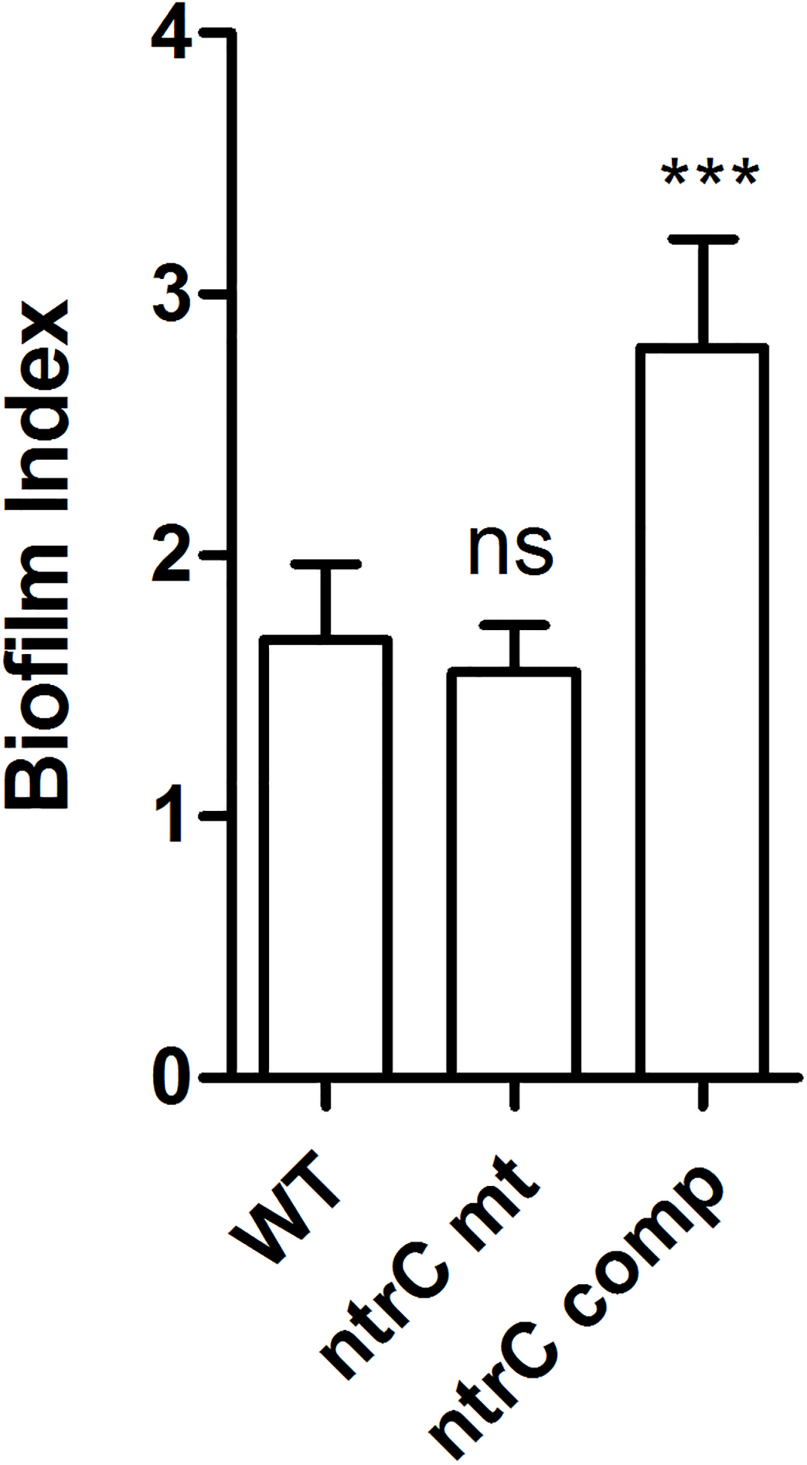
Biofilm formation is only slightly dependent on NtrC. Biofilm production was assessed for the wild type, *ntrC* mutant and complemented strain in 96-well plates. The columns represent the mean biofilm index generated from independent biological triplicate cultures of each strain. The error bars indicate the standard deviation. The increased biofilm formation in the complemented mutant compared to the wild type was statistically significant (*** p<0.0001). However, the slight reduction in the mutant was not statistically significant (ns).

The results from this comparative transcription analysis (Table 1 and S3 Table) suggest that NtrC is required for activation of transcription of the cepacian clusters (*bceI* and *bceII*). Cepacian is the main EPS produced by *B*. *cenocepacia* H111 [29, 45–47] and the expression of these two clusters has previously been shown to be induced under nitrogen limited conditions and controlled by σ^54^ [24]. Indeed, EPS production was reduced in the *ntrC* mutant to a similar extent as that of the σ^54^ mutant. EPS production was restored to the level of the wild-type in the complemented *ntrC* mutant (Fig 3). Biofilm formation, which was previously shown to be dependent on the alternative sigma factor (σ^54^) [24], was only slightly affected in the *ntrC* mutant. However, the complemented strain produced significantly more biofilm than the wild-type strain (Fig 4).

### NtrC regulates swarming and swimming motility

**Fig 5:**
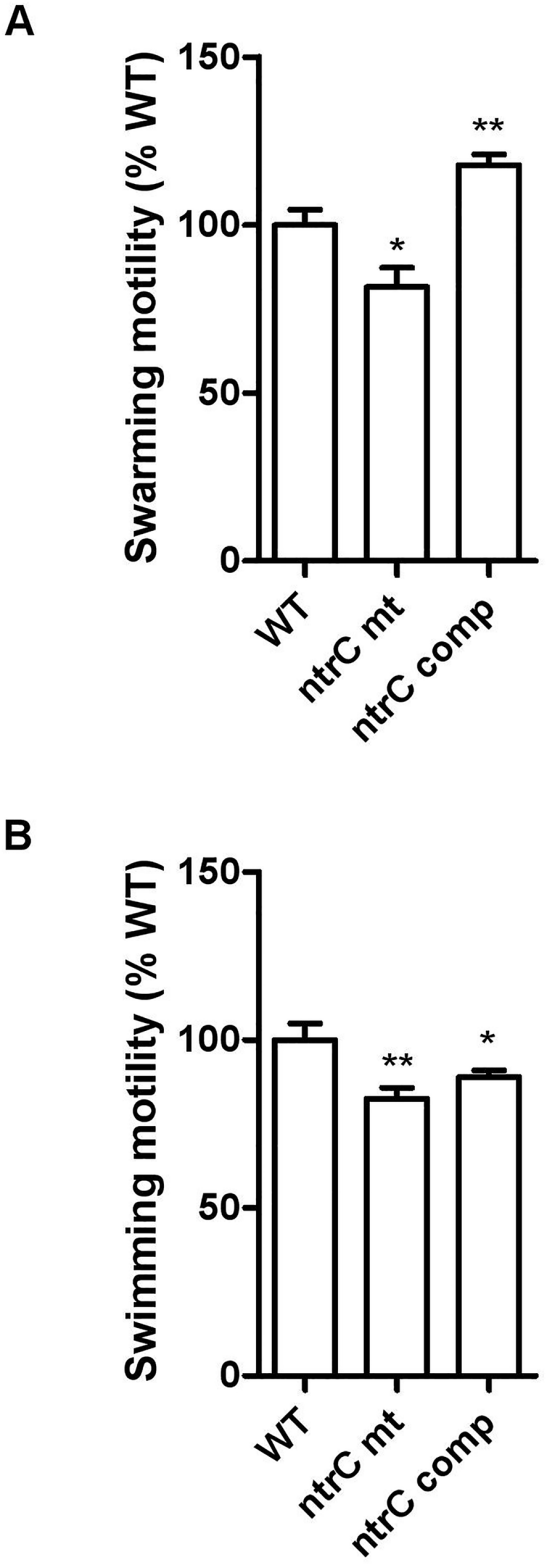
NtrC–dependent motility. The histograms show the swarming (A) and swimming (B) motility of the *ntrC* mutant and the complemented mutant relative to the wild-type strain H111. Both assays were performed in triplicate. Significance was calculated by comparing the mutant or the complemented mutant with the wild type (* p<0.05 and ** p<0.01).

The positive effect of NtrC on the transcription of genes encoding the flagellar motor (*motAB*) and flagellar biosynthesis proteins, prompted us to test motility of the strains. We first compared the swarming ability of the wild type strain with those of the *ntrC* mutant and the complemented strain (Fig 5A). The *ntrC* mutant was able to swarm, but to a lesser extent than wild type H111. Complementation increased swarming to above the level of the wild type. Swimming motility was also compromised in the *ntrC* mutant, but this defect was only partly restored in the complemented strain (Fig 5B). The results clearly show that NtrC plays an important role in the control of motility. The stronger effect on swarming is in line with the decreased transcription of *rhlA* (coding for a rhamnolipid) and motility-controlling flagellar-associated genes, such as *flgN* and *flgL*, in the *ntrC* mutant (Table 2).

### Virulence of *B*. *cenocepacia* H111 in the *C*. *elegans* infection model is not regulated by NtrC

Since σ^54^ was previously shown to be important for virulence in the *C*. *elegans* model, we set out to elucidate if this phenotype is also dependent on NtrC. For this, we fed nematode larvae with the wild type, the *ntrC* mutant and the complemented mutant. As a positive control, the σ^54^ mutant was included. While the σ^54^ mutant showed reduced virulence [24], worms infected with the *ntrC* mutant developed only to the L1-L2 larval stage, similarly to worms fed with the wild-type strain. This result is in line with our RNA-Seq data, which showed that the transcription of the nematocidal gene *aidA* (I35_7308), which was shown to be dependent on σ^54^ [24], was not controlled by NtrC (S3 Fig). The role of NtrC in the pathogenicity of H111 was also assessed in a different infection model, the larvae of the greater wax moth *Galleria mellonella*. In this model, we again did not observe significant attenuation of pathogenicity of the *ntrC* mutant compared with the wild type (data not shown).

## Discussion

We previously identified the *B*. *cenocepacia* genes responding to nitrogen starvation conditions and found that the alternative sigma factor σ^54^ (or RpoN) plays a major role in the control of nitrogen metabolism. That study furthermore uncovered that σ^54^ controls other important cellular processes such as EPS production, biofilm formation and *C*. *elegans* virulence [24]. Sigma 54 is an alternative sigma factor since it binds to a characteristic -24/-12 binding sequence (GGcacg-N4-ttGC) in the promoter of target genes and requires an additional ATP-dependent activation event to initiate transcription. This step is provided by transcriptional activators with an AAA (ATPase Associated with various cellular Activities) protein domain, which bind as inactive dimers to a consensus sequence upstream of the promoter, assemble as hexameric rings (in their active form) and interact through DNA looping with the σ^54^ promoter complex to activate transcription [20]. This requirement for an activator protein (or EBP) allows σ^54^ to tightly and rapidly control gene expression in response to cellular and extracellular signals that regulate the activity of a specific AAA-domain containing protein. Bacteria usually encode several AAA-family activator proteins and each one is needed for a specific and precise response to an environmental change. By looking for all *B*. *cenocepacia* proteins containing a σ^54^ activation AAA domain (PFAM family: PF00158), we were able to identify 22 proteins that could potentially serve as σ^54^ activator proteins (S4 Table). Among them we identified the regulatory protein NtrC, which is known to be the master regulator of nitrogen control in other bacteria. The *ntrC* gene had previously been shown to have increased expression under nitrogen limiting conditions [24]. The *ntrC* gene is usually located downstream of *ntrB*, which encodes a sensor kinase that phosphorylates the response regulator NtrC in nitrogen limited environments. In this study, we first performed a comprehensive growth analysis of an *ntrC* mutant on 95 different nitrogen sources including all common amino acids, the nitrogenous bases, several di-peptides and other compounds (Biolog Phenotype MicroArray). The *ntrC* mutant was revealed to be affected in the utilisation of 7 N sources i. e. nitrate, urea, L-citrulline, acetamide, DL-lactamide, allantoin and parabanic acid suggesting that utilisation of these sources in *B*. *cenocepacia* is dependent on the presence of a functional NtrC. We next used RNA-Seq analysis to elucidate the role of NtrC in the control of transcription during nitrogen starvation conditions. The results clearly show that NtrC is a major regulator under nitrogen limiting conditions and that the large majority of the NtrC-regulated genes are co-regulated by σ^54^. At the phenotypic level, the *ntrC* mutant strain behaved like a σ^54^ mutant strain for the utilization of alternative nitrogen sources such as nitrate and urea. Both urea and nitrate were not utilised as nitrogen sources by the mutant strain (Fig 1 and S2 Table). Accordingly, the transcription of genes involved in urea and nitrate assimilation (urease and nitrate/nitrite reductase, respectively) was significantly down-regulated in the *ntrC* mutant strain (Table 1). In addition to, and in line with, a poor utilization of allantoin in the *ntrC* mutant (S2 Fig and S2 Table), two genes coding for allantoicases (I35_0495 and I35_1962, Table 2 and S3 Table) showed significantly decreased expression in the *ntrC* mutant. Inspection of the NtrC regulon revealed two clusters of genes (*bce*I and *bce*II) involved in the production of cepacian, the main EPS in *B*. *cenocepacia* [29, 45–47]. Accordingly, EPS production was clearly reduced in an *ntrC* mutant and this defect could be rescued by genetic complementation. Control of EPS synthesis by NtrC has been demonstrated in several bacteria including the human pathogen *Vibrio vulnificus* [48], *Agrobacterium* sp. ATCC 31749 [49] and *Sinorhizobium meliloti* [50]. However, and in contrast to our previous results obtained with the σ^54^ mutant [24], biofilm formation was only slightly reduced in the *ntrC* mutant, suggesting that σ^54^ is using another EBP for controlling biofilm formation. This result also suggested that the reduced transcription of the *bce*I and *bce*II clusters does not drastically affect biofilm formation in microtiter plates.

We show here that the *ntrC* mutant is affected in motility and for the first time that the ability of *B*. *cenocepacia* H111 to swarm is controlled by NtrC and by σ^54^ (Fig 5). Many factors have been shown to regulate swarming [51, 52]. For example, in *Serratia liquefaciens* and in other bacteria, the *flhD* master operon needed for flagellar biosynthesis is essential for swarming motility [41, 53]. In *B*. *cenocepacia* H111 this social motility is under the control of the CepRI and RpfRF quorum sensing systems [40, 54] and in *B*. *glumae* it has recently been shown that quorum sensing controls swarming through the regulation of rhamnolipid biosynthesis under nutrient-limited conditions [55, 56]. In *Pseudomonas aeruginosa* swarming has been shown to be controlled at the post-transcriptional level by the RsmAB system and by c-di-GMP levels [57–59]. While transcription of *cepRI* and *rpfRF* was not altered in a *ntrC* mutant, we noted in our RNA-Seq data that NtrC positively regulates transcription of several genes which could be involved in the control of swarming motility: i) *motA* and *motB*, encoding the flagellar motor as well as several genes involved in flagellar biosynthesis; ii) genes involved in regulation of c-di-GMP levels by coding for proteins with a diguanylate cyclase phosphodiesterase (EAL) domain and iii) the gene *rhlA*, which encodes a 3-(3-hydroxyalkanoyloxy) alkanoic acid synthase, probably involved in rhamnolipid biosynthesis. Additional work will be required to test these possibilities.

The RNA-Seq approach allowed us to verify genes expected to be under NtrC control [60, 61] and to identify new potential NtrC targets such as the cepacian clusters and genes involved in motility (*motA*, *motB*, *flgL*, *flgK*, *flgN*, *rhlA*) (Table 1). Moreover, transcription of the holin and anti-holin gene pair *cidA* and *cidB* was significantly down-regulated in a *ntrC* mutant suggesting that this system, which is known to control peptidoglycan hydrolase activity and penicillin tolerance in *Staphylococccus aureus* [62] and has recently been proposed to be a key player in the regulation of the stress response [63], is under NtrC control in *B*. *cenocepacia* H111. However, our *ntrC* mutant was neither affected in antibiotic resistance nor in resistance to oxidative stress (data not shown).

Several genes coding for proteins involved in the TCA cycle were found among the genes showing increased transcription in the absence of NtrC under nitrogen limiting conditions, strengthening the proposal made by Hervas and co-workers that in *Pseudomonas putida*, NtrC represses carbon metabolism [61]. The fact that NtrC plays an important role in maintaining the balance between nitrogen and carbon metabolism under nitrogen limiting conditions may explain why constantly higher *ntrC* expression in the complemented mutant (driven by the promoter of the expression vector pBBR1MCS-5) leads to a growth defect in this strain.

This work demonstrates that NtrC is an activator of σ^54^-dependent gene transcription, that controls not only nitrogen metabolism but also various other functions, including EPS production and motility. However, as yet unidentified σ^54^ activators are required to control other phenotypes such as biofilm production and virulence to *C*. *elegans*. Further studies will be required to identify these activators.

## Supporting information

S1 Fig Growth of the *B*. *cenocepacia* H111 *ntrC* mutant in minimal medium containing citrate as carbon source was delayed compared to the wild type. Wild-type and *ntrC* mutant strains were grown in AB minimal medium from a starting OD_600_ of 0.05. Optical density was monitored over about 20 hours. The dotted line shows OD_600_ = 0.5, after which point the samples were subjected to nitrogen starvation and then harvested for RNA-Seq. The experiment was done in triplicate. Error bars indicate standard deviation.
(DOCX)

S2 Fig Differentially transcribed genes categorized by functional classification according to EggNOG. Percentages of induced and repressed genes are given for the comparison of *ntrC* mutant vs. wild-type cells grown under nitrogen limiting conditions. Percentages were calculated by dividing the number of significantly induced or repressed genes (Table 1) in each category by the total number of retained genes in the corresponding category. Asterisks (*) indicate statistical significance for overexpressed genes in a particular category (p-value < 0.01). C, energy production and conversion; E, amino acid transport and metabolism; F nucleotide transport and metabolism; G carbohydrate transport and metabolism; H coenzyme transport and metabolism; I lipid transport and metabolism; J translation, ribosomal structure and biogenesis; K transcription; L replication, recombination and repair; M cell wall/membrane/ envelope biogenesis; N cell motility; O post-translational modification, protein turnover and chaperon; P inorganic ion transport and metabolism; Q secondary metabolites biosynthesis, transport and catabolism; R general function prediction only; S function unknown; T signal transduction mechanisms; U intracellular trafficking, secretion and vesicular transport; V defense mechanisms.
(DOCX)

S3 Fig The virulence *B*. *cenocepacia* H111 to *C*. *elegans* is not dependent on NtrC. Pathogenicity assay of bacterial strains to *C*. *elegans* N2 strain was carried out as described in the material and methods. The number of L1 larvae in each well of a 96-well plate was counted after seeding with the bacterial strains to be tested. After 48 hours co-incubation at 20°C, the developmental stages of the worms were evaluated and the numbers were counted. Error bars represent standard deviation of the means (n = 3).
(DOCX)

S1 Table List of strains, constructs and primers used in this study. (DOCX)

S2 Table N source utilization of H111 wild-type and *ntrC* mutant strains assayed using Biolog PM3b plates, determined by measuring the OD_590_ in each well after 24 hours incubation at 37°C. (XLSX)

S3 Table List of the 400 top-ranked differentially transcribed genes in the *ntrC* mutant compared to the wild-type under nitrogen limited growth condition (DE-Seq analysis, p-value < 10^−13^, absolute log_2_(Fold Change) > 1.5). (XLSX)

S4 Table List of the 22 proteins encoded in the *B*. *cenocepacia* H111 genome with a σ^54^ activation AAA domain (PFAM family: PF00158). (XLSX)
