# Supplementary material for: NtrC-dependent control of exopolysaccharide synthesis and motility in Burkholderia cenocepacia H111
Source: PLoS One. 2017 Jun 29;12(6):e0180362. doi: 10.1371/journal.pone.0180362 (PMC5491218; doi:10.1371/journal.pone.0180362)
Supplement: S1 Fig — Wild-type and ntrC mutant strains were grown in AB minimal medium from a starting OD600 of 0.05. Optical density was monitored over about 20 hours. The dotted line shows OD600 = 0.5, after which point the samples were subjected to nitrogen starvation and then harvested for RNA-Seq. The experiment was done in triplicate. Error bars indicate standard deviation. (DOCX) [file pone.0180362.s001.docx]

**S1 Figure.**


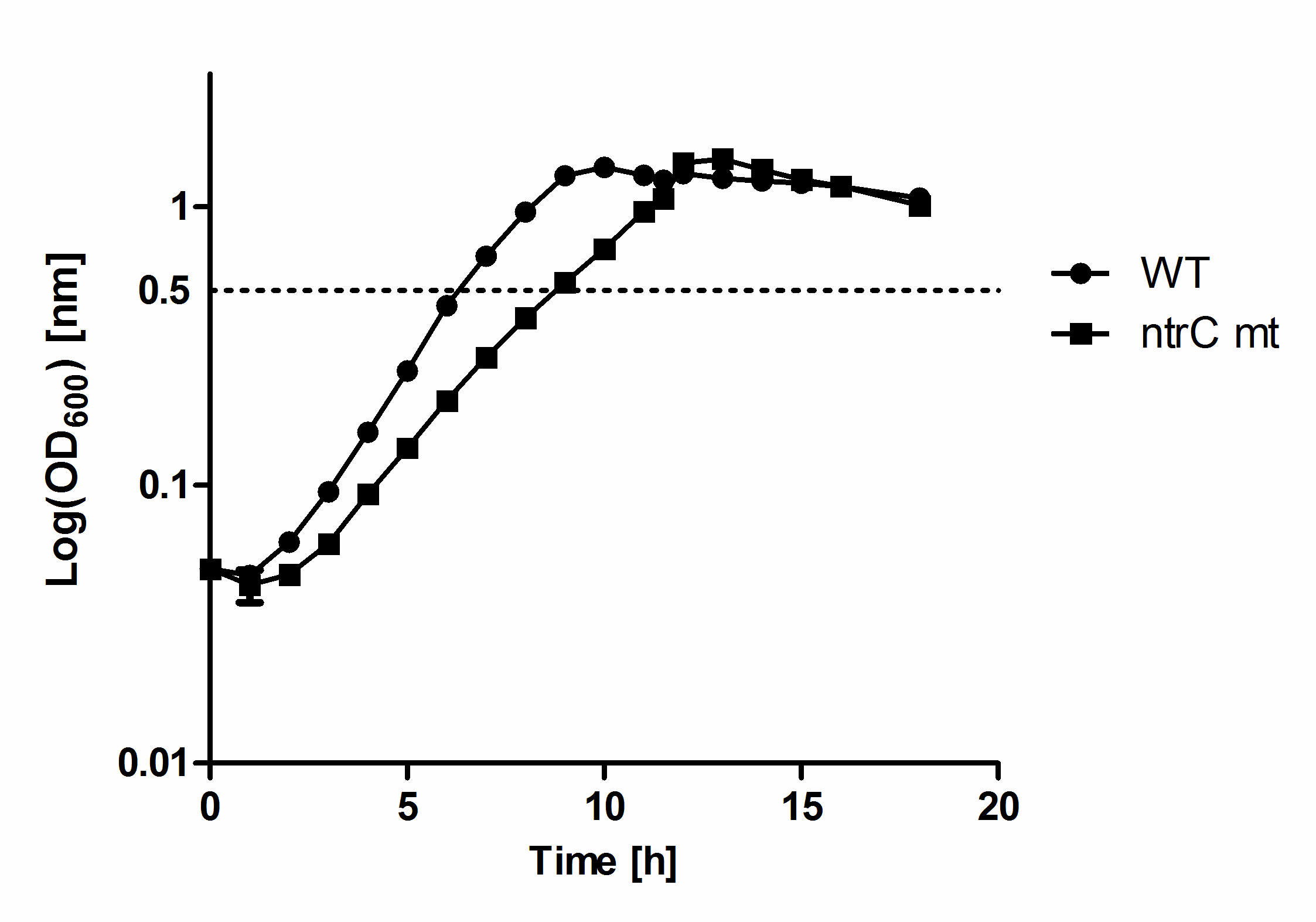


**S1 Fig. Growth of the *B. cenocepacia* H111 *ntrC* mutant in minimal medium containing citrate as carbon source was delayed compared to the wild type.** Wild-type and *ntrC* mutant strains were grown in ABC minimal medium start from OD_600_ at 0.05. Optical density was monitored over about 20 hours. The dotted line marked OD_600_ = 0.5, after which point the samples were subsequently subject to nitrogen starvation and then harvested for RNA-seq. The experiment was done in triplicate. Error bars indicate standard deviation.
