# Supplementary material for: NtrC-dependent control of exopolysaccharide synthesis and motility in Burkholderia cenocepacia H111
Source: PLoS One. 2017 Jun 29;12(6):e0180362. doi: 10.1371/journal.pone.0180362 (PMC5491218; doi:10.1371/journal.pone.0180362)
Supplement: S2 Fig — Percentages of induced and repressed genes are given for the comparison of ntrC mutant vs. wild-type cells grown under nitrogen limiting conditions. Percentages were calculated by dividing the number of significantly induced or repressed genes (Table 1) in each category by the total number of retained genes in the corresponding category. Asterisks (*) indicate statistical significance for overexpressed genes in a particular category (p-value < 0.01). C, energy production and conversion; E, amino acid transport and metabolism; F nucleotide transport and metabolism; G carbohydrate transport and metabolism; H coenzyme transport and metabolism; I lipid transport and metabolism; J translation, ribosomal structure and biogenesis; K transcription; L replication, recombination and repair; M cell wall/membrane/ envelope biogenesis; N cell motility; O post-translational modification, protein turnover and chaperon; P inorganic ion transport and metabolism; Q secondary metabolites biosynthesis, transport and catabolism; R general function prediction only; S function unknown; T signal transduction mechanisms; U intracellular trafficking, secretion and vesicular transport; V defense mechanisms. (DOCX) [file pone.0180362.s002.DOCX]

**S2 Figure.**


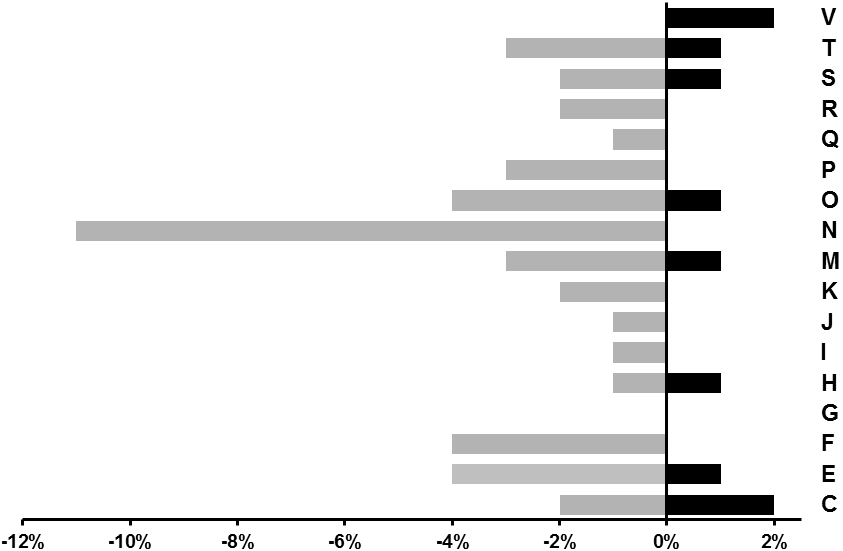


Repressed

Induced

*

*

*

**S2 Fig. Differentially transcribed genes categorized by functional classification according to EggNOG.** Percentages of induced and repressed genes are given for the comparison of *ntrC* mutant vs. wild-type cells growing under nitrogen limiting conditions. Percentages are calculated by dividing the number of significantly induced or repressed genes (Table 1) in each category by the total number of retained genes in the corresponding category. Asterisks (*) indicate statistical significance for overexpressed genes in a particular category (p-value < 0.01). C, energy production and conversion; E, amino acid transport and metabolism; F nucleotide transport and metabolism; G carbohydrate transport and metabolism; H coenzyme transport and metabolism; I lipid transport and metabolism; J translation, ribosomal structure and biogenesis; K transcription; L replication, recombination and repair; M cell wall/membrane/ envelope biogenesis; N cell motility; O post-translational modification, protein turnover and chaperon; P inorganic ion transport and metabolism; Q secondary metabolites biosynthesis, transport and catabolism; R general function prediction only; S function unknown; T signal transduction mechanisms; U intracellular trafficking, secretion and vesicular transport; V defense mechanisms.
