# Supplementary material for: NtrC-dependent control of exopolysaccharide synthesis and motility in Burkholderia cenocepacia H111
Source: PLoS One. 2017 Jun 29;12(6):e0180362. doi: 10.1371/journal.pone.0180362 (PMC5491218; doi:10.1371/journal.pone.0180362)
Supplement: S3 Fig — Pathogenicity assay of bacterial strains to C. elegans N2 strain was carried out as described in the material and methods. The number of L1 larvae in each well of a 96-well plate was counted after seeding with the bacterial strains to be tested. After 48 hours co-incubation at 20°C, the developmental stages of the worms were evaluated and the numbers were counted. Error bars represent standard deviation of the means (n = 3). (DOCX) [file pone.0180362.s003.docx]

**S3 Figure.**


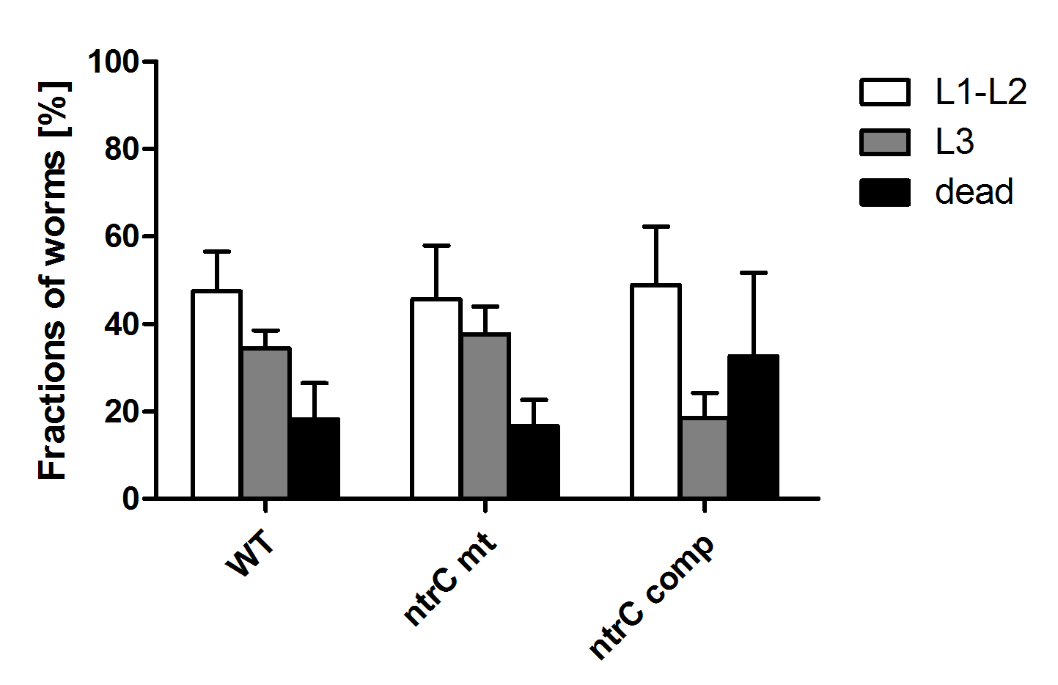


**S3 Fig. The virulence *B. cenocepacia* H111 to *C. elegans* is not dependent on NtrC.** Pathogenicity assay of bacterial strains to *C. elegans* N2 strain was carried out as described in the material and methods. The number of L1 larvae in each well of a 96-well plate was counted after seeding with the bacterial strains to be tested. After 48 hours co-incubation at 20°C, the developmental stages of the worms were evaluated and the numbers were counted. Error bars represent standard deviation of the means (n=3).
