# Supplementary material for: NtrC-dependent control of exopolysaccharide synthesis and motility in Burkholderia cenocepacia H111
Source: PLoS One. 2017 Jun 29;12(6):e0180362. doi: 10.1371/journal.pone.0180362 (PMC5491218; doi:10.1371/journal.pone.0180362)
Supplement: S1 Table — (DOCX) [file pone.0180362.s004.docx]

| **S1 Table**. List of strains, constructs and primers used in this study | |  |
| --- | --- | --- |
|  |  |  |
| **Strain or plasmid** | **Description** | **Reference** |
| Strains |  |  |
| *E. coli* |  |  |
| cc118λ-pir | Δ(ara-leu) *araD* Δ*lacX74 galE galK phoA20 thi1 rpsE rpoB argE(Am) recAl* λ pir; Strep^R^ | [1] |
| Top10 | Δ*lacX74 ara*Δ*139*Δ*(ara-leu)* | Invitrogen |
| *B. cenocepacia* |  |  |
| H111 | CF isolate from Germany, genomovar III | [2,3] |
| H111-ntrC_Bc_ | *ntrC*_Bc_::pSHAFT2 mutant of H111; Cm^R^ | This study |
| H111-ntrC_Bc_, pBBR*ntrC* | *ntrC*_Bc_::pSHAFT2 mutant of H111 complemented with pBBR1-*ntrC*; Cm^R^ and Gm^R^ | This study |
| H111-rpoN_Bc_ | *rpoN*_Bc_::pSHAFT2 mutant of H111; Cm^R^ | [4] |
| H111-rpoN_Bc_, pBBR*rpoN* | *rpoN*_Bc_::pSHAFT2 mutant of H111 complemented with pBBR1-*rpoN*; Cm^R^ and Gm^R^ | [4] |
|  |  |  |
| Plasmids |  |  |
| pGEM-T Easy | Cloning vector; Amp^R^, *lac*Z | Promega |
| pRK2013 | Helper plasmid; Km^R^ | [5] |
| pSHAFT2 | Broad-host-range suicide plasmid, mobilisable for conjugation; Cm^R^ | [6] |
| pBBR1MCS-5 | Broad host-range cloning vector; Gm^R^ | [7] |
| pSHAFT2-*ntrC* | pSHAFT2 containing an internal fragment of I35_2149 (*BCAL2222*) for mutagenesis; Cm^R^ | This study |
| pBBR1-*ntrC* | pBBR1MCS-5 containing I35_2149 (*BCAL2222*); Gm^R^ | This study |
|  |  |  |
| Oligonucleotides |  |  |
| ntrC_mut_new_F | AAGGACCTGCTCGAATCTGA | This study |
| ntrC_mut_new_R | CCATCACGGTCAGCCAGT | This study |
| ntrC_Comp_F | CACCCATATCTGACCGACCT | This study |
| ntrC_Comp_R | AGGATTCGGACATGGAGGAT | This study |
| ntrC_I35_2149_F | GAAGGACCTGCTCGAATCTG | This study |
| ntrC_I35_2149_R | ATAGAACTGCCCGTCCGACA | This study |
| ureC_I35_0767_F | TTGAAACTGCACGAGGACTG | This study |
| ureC_I35_0767_R | ACCTTCGGTGTGGTACGTGT | This study |
| glnA_I35_2151_F | ATGAAGAGTCGACCCTCGTG | This study |
| glnA_I35_2151_R | GACATGTCCGTGTTCCACTG | This study |
| bceE_I35_4771_F | GACGAAGCCTGAAGTGTTCC | This study |
| bceE_I35_4771_R | TGATTTGCCGCATGTAGAAG | This study |
| I35_4928_F | CTTTCAGCGGATCGTCTCTG | This study |
| I35_4928_R | GAACGGGAAGGTCAGGTAGC | This study |
| rhlA_I35_6233_F | GCCAGACCGTCCAGTATCTC | This study |
| rhlA_I35_6233_R | CGAAGTGCTCGACCAGGTA | This study |
| motA_I35_0133_F | AGCTGATGGCGCTTCTTTAC | This study |
| motA_I35_0133_R | TCCATCAGGCTTTCGATCTC | This study |
| flgL_I35_3089_F | AGATGAACGTCGCCCAGAT | This study |
| flgL_I35_3089_R | AGCGTCTGGTCTTCCTTCTG | This study |
| flgN_I35_3103_F | GGATCGTCGAGAAGAAAAGC | This study |
| flgN_I35_3103_R | GGATCAGCATTCCGTTGTTC | This study |
| rpoD_I35_4837_F | AGGAAGCCGAAGAGGAAGAG | This study |
| rpoD_I35_4837_R | GTCGAACCACTCGCTGATCT | This study |
| pSHAFT_R | ACATGTGGAATTGTGAGC | This study |
|  |  |  |

**References**

1. Herrero M, de Lorenzo V, Timmis KN. Transposon vectors containing non-antibiotic resistance selection markers for cloning and stable chromosomal insertion of foreign genes in gram-negative bacteria. J Bacteriol. 1990;172: 6557–6567. Available: http://www.ncbi.nlm.nih.gov/pubmed/2172216

2. Romling U, Wingender J, Muller H, Tummler B. A major *Pseudomonas aeruginosa* clone common to patients and aquatic habitats. Appl Env Microbiol. 1994;60: 1734–1738.

3. Gotschlich A, Huber B, Geisenberger O, Togl A, Steidle A, Riedel K, et al. Synthesis of multiple N-acylhomoserine lactones is wide-spread among the members of the *Burkholderia cepacia* complex. Syst Appl Microbiol. 2001;24: 1–14. doi:10.1078/0723-2020-00013

4. Lardi M, Aguilar C, Pedrioli A, Omasits U, Suppiger A, Cárcamo-Oyarce G, et al. σ54-dependent response to nitrogen limitation and virulence in *Burkholderia cenocepacia* H111. Appl Environ Microbiol. 2015; doi:10.1128/AEM.00694-15

5. Phadnis SH, Berg DE. Identification of base pairs in the outside end of insertion sequence IS50 that are needed for IS50 and Tn5 transposition. Proc Natl Acad Sci U S A. 1987;84: 9118–9122. Available: http://www.ncbi.nlm.nih.gov/pubmed/2827168

6. Shastri S, Spiewak HL, Sofoluwe A, Eidsvaag VA, Asghar AH, Pereira T, et al. An efficient system for the generation of marked genetic mutants in members of the genus *Burkholderia*. Plasmid. 2017;89: 49–56. doi:10.1016/j.plasmid.2016.11.002

7. Kovach ME, Elzer PH, Steven Hill D, Robertson GT, Farris M a., Roop RM, et al. Four new derivatives of the broad-host-range cloning vector pBBR1MCS, carrying different antibiotic-resistance cassettes. Gene. 1995;166: 175–176. doi:10.1016/0378-1119(95)00584-1
